# Preliminary Report on the Acquisition, Persistence, and Potential Transmission of *Citrus tristeza virus* by *Diaphorina citri*

**DOI:** 10.3390/insects12080735

**Published:** 2021-08-17

**Authors:** Fengnian Wu, Mochi Huang, Eduardo G. P. Fox, Jiaquan Huang, Yijing Cen, Xiaoling Deng, Meirong Xu

**Affiliations:** 1 Guangdong Province Key Laboratory of Microbial Signals and Disease Control/Citrus Huanglongbing Research Laboratory, South China Agricultural University, Guangzhou 510642, China; scau100@163.com (F.W.); huangmochi1226@163.com (M.H.); jiaquan_terry@stu.scau.edu.cn (J.H.); cenyj@scau.edu.cn (Y.C.); 2 School of Food Engineering and Biotechnology, Hanshan Normal University, Chaozhou 521041, China; 3 Instituto de Biofísica Carlos Chagas Filho, Centro de Ciências da Saúde, Universidade Federal do Rio de Janeiro, Rio de Janeiro 21941-902, Brazil; ofoxofox@gmail.com

**Keywords:** *Citrus tristeza virus*, acquisition and transmission, Huanglongbing, psyllid

## Abstract

**Simple Summary:**

*Citrus tristeza virus* (CTV) is the causal agent of one of the most serious diseases of citrus and is described to be vectored by several aphid species. There have been no published reports of either acquisition or transmission of CTV by other insects, including phloem-feeding sternorrhynchans. The Asian citrus psyllid *Diaphorina citri* is an economically important pest since it is the vector of the bacterium associated with Huanglongbing (HLB) in citrus crops. We hereby reported the detection of CTV from field-collected *D. citri* and estimated the ability of these insects to acquire and transmit the virus. Under controlled conditions, *D. citri* nymphs were shown to acquire CTV from citrus trees, and the virus persisted in the psyllids for over 15 days. Controlled experiments also suggest that *D. citri* transmit CTV to healthy citrus plants but not to orange jasmine plants, a favorite host of *D. citri*. The results indicate *D. citri* is a potential vector of pathogens for two major citrus diseases: HLB and Citrus tristeza.

**Abstract:**

*Citrus tristeza virus* (CTV) is one of the most important citrus tree viruses: a graft-transmissible virus that can be vectored by several aphid species. *Diaphorina citri* is the insect vector of “*Candidatus* Liberibacter spp.”, a bacterium associated with citrus Huanglongbing (HLB). However, no detailed description of the relationship between CTV and *D. citri* has been reported. In this study, *D. citri* adults collected from CTV-infected “Shatangju” mandarin, “Newhall” sweet orange, and “fingered citron” trees in different orchards yielded CTV-positive rates of 40%, 65%, and 95%, respectively, upon detection by conventional PCR. Illumina HiSeq sequencing followed by *de novo* assembly recovered the primary full CTV genome from the RNA of 30 *D. citri* adults sampled from CTV-positive citrus plants. Molting and adult emergence did not affect the presence or titers of CTV within the *D. citri*; however, the persistence of CTV in psyllids varied among different host plant species. Groups of 10 *D. citri* (from a population 85% CTV-positive) were shown to potentially transmit CTV to two citrus species, “Shatangju” mandarin and “Eureka” lemon, yielding 58.33% and 83.33% CTV-positive plants, respectively. No transmission of CTV to orange jasmine plants occurred. Thus, this study reports on the ability of *D. citri* to acquire and transmit CTV, making *D. citri* as a vector of two important citrus pathogens, warranting further attention and investigation.

## 1. Introduction

Among the common viral diseases of citrus, the globally distributed citrus tristeza is one of the most economically important, associated with *Citrus tristeza virus* (CTV). Tristeza kills hundreds of millions of citrus trees worldwide, nowadays mainly in South Africa (since 1910), Argentina and Brazil (since 1970), and the U.S. (since 1950), affecting especially sweet orange (*Citrus* × *aurantium* var. *sinensis* L.) and mandarin (*C. reticulata* Blanco) grafted on sour orange (*C*. × *aurantium* var. *aurantium* L.) rootstocks [1,2]. As an example, the incidence of this disease among sweet orange and mandarin grafted on sour orange rootstocks escalated from 11% in 1989 to 53% in 1998, and then to almost 90% in 2000 in the Valencian community of Spain [3]. Some CTV isolates are reported to be associated with the citrus quick decline disease [4]. Some other isolates can cause stem pitting, which results in a significant drop in the fruit quality and yield of some citrus species [2,5]. Natural CTV hosts include species of the genus *Citrus*, but the virus has also been detected in other experimentally inoculated related genera in the Rutaceae family *Aegle*, *Aeglopsis*, *Afraegle*, *Atalantia*, *Citropsis*, *Clausena*, *Hesperethusa*, *Merrillia*, *Pamburus*, and *Pleiospermium* [2,6,7].

Citrus tristeza has been recorded as a widely distributed citrus disease in China since 1979, but it was not taken seriously because the local citrus species varieties (most of which mandarins) and their rootstocks were believed CTV-tolerant [8]. With the recent rapid expansion of the citrus industry, CTV has become a growing concern, with the affected trees exhibiting stunted growth, stem pitting, yield loss, and poor fruit quality [2,9]. The incidence of CTV was estimated to be as high as 60% among citrus orchards around the provinces of Fujian, Guangdong, Jiangxi, Hubei, and Hunan from the 1980s to the 1990s [10,11]. About 30 years ago, almost all of the sweet orange orchards in Zhejiang Province were found to be CTV-positive [10]. In three pomelo groves in Sichuan Province, about 20% to 30% of trees were recorded as infected, based on identified symptoms in 1992. However, the rates of trees presenting citrus tristeza disease symptoms increased to as much as 80% in 1997 [11]. Severe damage was recorded among sweet oranges on trifoliate orange (*Poncirus trifoliata* (L.) Raf.) rootstocks in Chongqing city in 1998, and sweet oranges on *C. medica* L. rootstocks in Binchuan and Jianshui (Yunnan Province) [10,12]. CTV incidences ranging from 26.4% to 61.4% were recorded in sweet orange orchards in Gannan of Jiangxi Province in 2009 by surveying symptoms across the field sites and through enzyme-linked immunosorbent assay (ELISA) [13].

Belonging to the genus *Closterovirus* in the Closteroviridae family, CTV is a long flexuous virus (2,000 nm by 10 to 12 nm), currently the largest reported plant RNA virus (19.3 kb) [14]. CTV is a (+) single-stranded RNA (ssRNA) virus that includes 12 open reading frames (ORFs) that potentially code for 11 genes [2,15,16]. CTV is considered genetically diverse, with seven described strains differing in their nucleotide sequences by 10% to 20% [17]. Several methods have been developed to detect CTV, including ELISA procedures [18], conventional and fluorescence quantitative real-time polymerase chain reaction (PCR) assays [19].

CTV limits itself to the phloem and phloem-associated cells in citrus trees and is known to be graft-transmissible via CTV-positive buds [20]. It can be acquired and spread by several species of aphids in a semi-persistent manner. The aphids reported to vector CTV include *Aphis* (*Toxoptera*) *citricidus* (Kirkaldy), *Aphis gossypii* Glover, *Aphis spiraecola* Patch*, Aphis* (*Toxoptera*) *aurantii* (Boyer de Fonscolombe), and *Myzus persicae* (Sulzer) (Hemiptera: Sternorrhyncha: Aphididae)*,* where the pathogens are acquired in a non-circulative, semi-persistent mode, resulting in variable transmission efficiency [14,21,22,23,24]. Furthermore, CTV isolates influenced the experimental transmission efficiency by *Aphis* (*Toxoptera*) *citricidus*, the most efficient aphid vector [13]. To our knowledge, there are no published records of insects other than aphids vectoring CTV.

The Asian citrus psyllid (ACP), *Diaphorina citri* Kuwayama (Hemiptera: Sternorrhyncha: Liviidae), is considered to be one of the most important global pests of citrus because it transmits “*Candidatus* Liberibacter spp.”, uncultured alpha-proteobacteria associated with the citrus Huanglongbing (HLB) [25,26]. This psyllid was first recorded as a vector of HLB in Asia in 1967 and was demonstrated to transmit “*Ca.* Liberibacter asiaticus” (CLas) in China in 1977 [27,28,29,30]. CLas is transmitted by *D. citri* in a persistent-propagative manner [31,32,33]. Globally, HLB is a devastating disease of citrus crops that is managed chiefly through quarantine, production of pathogen-free nursery stocks, removal of infected trees from orchards, and control of *D. citri* populations [26,34]. *Diaphorina citri* has spread from South Asia to West Asia, Southeast Asia, parts of Australasia, and the Americas [35]. This psyllid is a phloem-feeding insect associated with the genus *Citrus* and some other Rutaceae, and its favorite plant host of *D*. *citri* is orange jasmine [36,37]. Nouri et al. performed a viral metagenome analysis of sequenced small RNAs and transcriptomes coupled with bioinformatics and found that virus-like sequences within *D. citri* were highly diverse but reported no known plant viral pathogens [38]. In a previous study, CTV was used as a vector carrying sequences for *D*. *citri* genes in RNAi research [39]. Given that *D. citri* is considered an important pest of citrus orchards, we wondered whether this psyllid would be able to carry other phytopathogens in addition to “*Candidatus* Liberibacter spp.”.

Reanalysis of raw data in RNAseq samples of *D. citri* from our previous study [40] had recovered some fragments of the CTV genome, which prompted the present investigation. We wondered whether HLB and citrus tristeza disease could be transmitted by the same vector. Therefore, the main objective of this study was to investigate the possibility that *D. citri* may carry, persist with, and ultimately transmit CTV under controlled conditions.

## 2. Materials and Methods

### 2.1. CTV Orchard Surveys

The presence of CTV in mature trees and *D.* *citri* populations was surveyed in three orchards, two in Qingyuan (23.67° N, 112.89° E) and Zhaoqing (23.32° N, 112.21° E), in Guangdong Province and one in Ganzhou (25.29° N, 114.92° E) of Jiangxi Province. The 8-year-old “Shatangju” mandarin trees were sampled at Qingyuan in May 2015, 5-year-old “fingered citron” (*C*. *medica* L. var. *sarcodactylis* Swingle) trees were sampled at Zhaoqing in July 2019, and 12-year-old sweet orange trees were sampled at Ganzhou in June 2016 (Table S1). All of the investigated orchards had over 2000 citrus plants. Local pest management among these orchards was 8–12 times per year. Twenty *D. citri* adults from different trees and 20 citrus trees were sampled from each orchard. Total RNA was extracted from the samples and assayed for the presence of CTV by conventional PCR.

After confirming that citrus trees and psyllids from all of the three orchards were CTV-positive, 60 4th-instar psyllids were collected from each of the orchards and kept on the original detached CTV-positive young shoots inside controlled environment chambers (with shoot inserted into moistened centrifuge tubes, inside Falcon^®^ tubes modified to accommodate the centrifuge tubes). To check whether CTV would persist through molts, 20 late-stage 4th instars (i.e., bound to molt in 24 h) from each of three chambers were collected, ten were used for RNA extraction, and the other ten were transferred to detached CTV-negative orange jasmine young shoots until the 4th instars had molted to the 5th instar. Newly molted 5th instars were collected, and RNA from each nymph was extracted. The RNA samples were reverse transcribed to cDNA and then used for CTV detection by reverse transcription real-time quantitative PCR (RT-qPCR). With a similar goal, the same procedure as above was employed by transferring 20 late-stage 5th instars to CTV-negative orange jasmine and testing ten late-stage 5th instars and ten newly emerged adults.

### 2.2. Plant and Insect Materials under Laboratory Conditions

As shown in Table S1, plants comprising “Shatangju” mandarin (*Citrus reticulata* Blanco “Shatangju”) scions grafted on trifoliate orange, “Eureka” lemon (*Citrus* × *limon* var. limon (L.) Burm. f.) grafted on rough lemon (*Citrus jambhiri* Lush), and orange jasmine (*Murraya paniculata* (L.) Jack) seedings were maintained in a screenhouse at South China Agricultural University (SCAU; 23.16° N, 113.36° E), Guangzhou, China. The two citrus species were reported in previous studies to have different tolerance to infection by CTV, and orange jasmine was reported resistant to CTV [41,42,43]. All plants were 2 to 3 years old with an average height of around 50 cm. All plants were confirmed CTV-negative by PCR and ELISA, as described further below. Concomitantly, buds of CTV-positive “Shatangju” mandarin trees from Zhaoqing (23.75° N, 112.24° E), Guangdong, were grafted onto CTV-negative “Shatangju” mandarin plants maintained in a separate screenhouse, and were used to rear CTV-positive psyllids (Table S1).

Adult *D. citri* collected from orange jasmine plants at SCAU campus were used to establish psyllid colonies on orange jasmine plants in a temperature-regulated greenhouse (25 ± 1 °C, 60% ± 2% relative humidity (RH)). The psyllid population had been reared for over 10 generations. Ten *D*. *citri* adults from the orange jasmine plants were tested individually by RT-qPCR every two weeks to ensure that psyllids colonies remained CTV-negative throughout the experiment.

### 2.3. Acquisition and Persistence of CTV in D. citri

**Figure 1:**
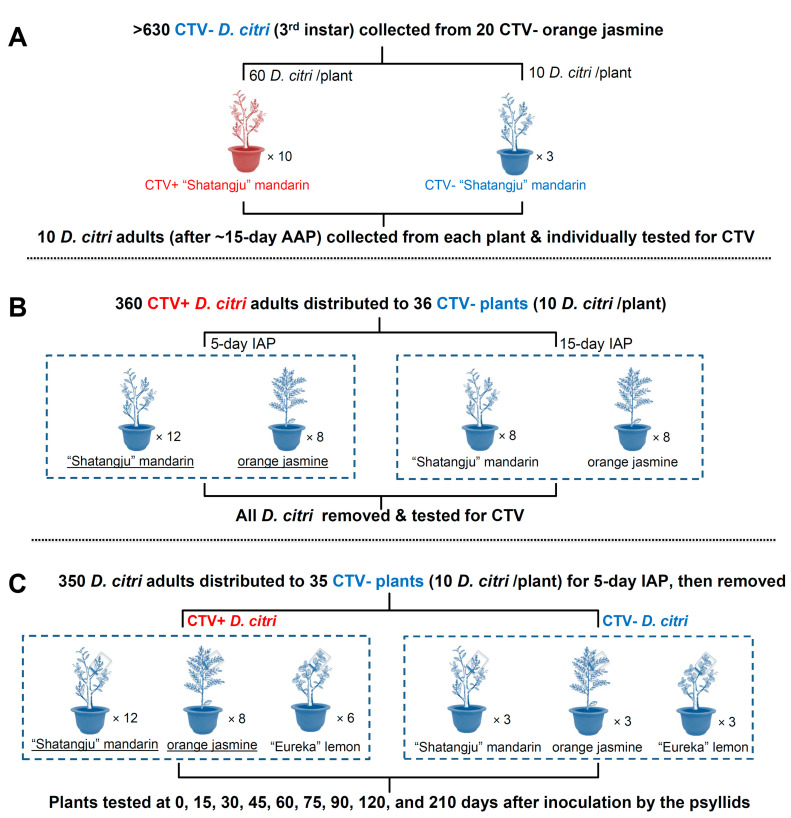
Experimental settings for testing acquisition of *Citrus tristeza virus* (CTV) by *Diaphorina citri* (**A**), viral persistence period within the psyllids (**B**), and CTV transmission capacity to healthy plants by *Diaphorina citri* (**C**). Groups of plants in figures B and C with their names underlined were the same used throughout the experiment. Groups highlighted in red were confirmed CTV-positive prior to each experimental step, and those highlighted in blue were confirmed CTV-negative prior to each experimental step. Legend: CTV+: CTV-positive; CTV-: CTV-negative; “Shatangju” mandarin: C*itrus reticulata* Blanco “Shatangju”; “Eureka” lemon: *Citrus* × *limon* var. *limon* (L.) Burm. f.; orange jasmine: *Murraya paniculata* (L.) Jack.; CTV+ *D. citri*: a group of *D. citri* with ~85% of them were CTV-positive; AAP: acquisition access period; IAP: inoculation access periods; 0 days: tested immediately after inoculation by psyllids.

The experimental design of this phase was based on the outcome of CTV persistence assessments of nymphs after molting and emergence. About 700 CTV-negative 3rd-instar *D. citri* were collected from 20 CTV-free orange jasmine plants, maintained as above. The nymphs were divided into 10 groups of 60 individuals. Each group of 60 was placed on one of 10 CTV-positive “Shatangju” mandarin plants (Figure 1A). One week after the emergence of adult *D*. *citri* (after an approximately 15-day-acquisition access period (AAP), when most nymphs had passed through 4th and 5th instars to become adults), ten adults from each tree were collected and tested for CTV titers individually. As shown in Figure 1B, the remaining *D*. *citri* adults from the 10 CTV-positive “Shatangju” mandarin plants were collected, randomly subdivided into groups of 10, and then each group was transferred to each of 20 CTV-negative “Shatangju” mandarin plants and to each of 16 CTV-negative orange jasmine plants for 5- or 15-day inoculation access periods (IAP).

After the 5-day IAP, all *D. citri* were collected from 12 of the 20 “Shatangju” mandarin and 8 of the 16 orange jasmine plants. After the 15-day IAP, all psyllids were collected from the remaining 8 “Shatangju” mandarin and 8 orange jasmine plants (Figure 1B). Adults of *D. citri* from three CTV-negative “Shatangju” mandarin or three CTV-negative orange jasmine plants were used as negative controls (Figure 1A). RNA was extracted from all collected *D. citri* adults individually and reverse transcribed into cDNA.

The CTV infection status of the initially CTV-negative “Shatangju” mandarin and orange jasmine plants were assessed using qPCR immediately after the 5-d or 15-d IAP. In addition, the “Shatangju” mandarin trees and orange jasmine plants exposed to CTV-positive *D*. *citri* for 5 days were also used for the next experiment about transmission.

### 2.4. Transmission of CTV by D. citri to Mandarin, Lemon, and Orange Jasmine Plants

The preliminary studies about the transmission capacity of CTV by *D. citri* were described under laboratory conditions using three species of the Rutaceae family with different tolerance to CTV. After a 15-day AAP, ten *D. citri* adults were transferred to a single shoot in each of 12 “Shatangju” mandarin trees, six “Eureka” lemon trees, and eight orange jasmine plants for a 5-day IAP. All 26 plants were tested CTV-negative by both qPCR and ELISA as described below. The psyllids were enclosed in meshed plastic gauze bags (250 mm(L) × 180 mm(W)) without touching the leaves. After the IAP, all psyllids and eggs together with their enclosing bags were removed. All plants were maintained in plant growth chambers (Jiangnan Instrument Company, RXZ-380A, internal dimensions 50 cm L × 50 cm W × 100 cm H), with three trees in each chamber, at 25 ± 1 °C, 65% ± 2% RH, 14:10 h L:D, 8000 lx (Figure 1C) during the IAP but were moved to the screenhouse after IAP. Leaves of approximately the same age were selected immediately following the IAP and at 15, 30, 45, 60, 75, 90, 120, and 210 days after IAP. Three leaves (= replicates) from each of three shoots, with one of them fed by the psyllids, from each plant were sampled. RNA was extracted from the three-leaf midribs individually. Any samples that tested positive by conventional PCR were further confirmed by RT-qPCR and ELISA. CTV-negative “Shatangju” mandarin, “Eureka” lemon plants, and orange jasmine plants, after exposure to CTV-negative *D. citri* adults, were used as negative controls for CTV detection (Figure 1C). Leaf and stem symptoms of CTV-positive plants were checked monthly (30 days) for 210 days. The barks from three branches of each tree were peeled to check the stem pitting status at 210 days after IAP. At the end of the experiment, crown diameter and height of CTV-positive and CTV-negative mandarin and lemon plants were measured with a measuring tape.

### 2.5. RNA Extraction and cDNA Synthesis

The RNA from each *D. citri* was extracted individually using TRlzol^®^ Reagent (Life Technologies, Guangzhou, China). RNA from the midribs of either citrus leaves or orange jasmine leaves was isolated using the TransZol Plant kit (TransGen Biotech, Beijing, China). Genomic DNA contamination was eliminated by digestion with RNase-free DNase I (TaKaRa, Shuzo, Kyoto, Japan). Total RNA concentration was estimated with fluorescence-based Qubit™ (Shanghai, China). The purity of total RNA was determined by absorbance using NanoDrop™ One (Thermo Scientific, Shanghai, China), where RNA samples with OD260/OD230 2.0–2.4 and OD260/OD280 of 1.8−2.2 were further reverse transcripted to cDNA. cDNA was synthesized from 500 ng of total RNA using Verso cDNA Synthesis (TransScript) kit (TransGen Biotech, Beijing, China) with random hexamers primers following the manufacturer’s instructions. The obtained cDNA samples were used as templates for PCR or qPCR amplification, as described below.

### 2.6. CTV Detection

Four methods were used for CTV detection to ensure genome integrity and reliability of our results. The methods were: conventional PCR (using conserved regions of the CTV genome for rapid detection while avoiding false negatives), RT-qPCR (enabling absolute quantification of CTV because of greater sensitivity and calculability [19,44]), CTV genome detection (ensuring genome integrity), and ELISA (detection of CTV viral protein coat). The results of these tests were standards for labeling samples as CTV- “positive” or “negative”, elsewhere in this manuscript. Each method is detailed below.

#### 2.6.1. Conventional PCR

Because of the high genetic diversity among known CTV isolates, a universal primer set (Uni-f: 5’-ATG TTA GCT AGA CGT CAA GGT T-3’/ Uni-r: 5’-TTC CCA AGC TGC CTG ACA TT-3’) (size of PCR product = 822 bp) for conventional PCR was designed based on the complete conserved sequence region of *p27* gene from 61 published CTV genomes downloaded from the GenBank nucleotide database (accessed on 20 July 2020) using Primer3web v. 4.1.0 [45]. Conventional PCR was performed with PrimeSTAR^®^ GXL DNA Polymerase (Takara, Shiga, Japan) under the following conditions: initial denaturation for 10 s at 98 °C, followed by 35 denaturation cycles of 10 s at 98 °C, annealing for 15 s at 54 °C, elongation for 1 min at 68 °C, and a 7-min long final extension step at 72 °C. Amplicon bands were excised, cleaned, and cloned into pEASY-T1 (TRANS, Beijing, China) vectors, and the resulting *Escherichia coli* plasmids carrying CTV fragments were extracted using AxyPrep Multisource Genomic DNA Miniprep kit (Axygen, Suzhou, China). The recombinant plasmids were purified using QIAprep Spin Miniprep Kit (QIAGEN, Germany) and sequenced with an ABI 3130 DNA sequencer (ABI, Foster, CA, USA) using the primer set M13F (5′-TGT AAA ACG ACG GCC AGT-3′) /M13R (5′-CAG GAA ACA GCT ATG ACC-3′). Amplicon sequences obtained were analyzed with BLAST in NCBI.

#### 2.6.2. Reverse Transcription Real-Time Quantitative PCR (RT-qPCR)

For absolute quantification using Taqman™ qPCR, CTV was detected using the primer set (qP20-f: 5′-TAT AGA GGC GAA ACT GCG AAT-3′/ qP20-r: 5′-CCT CAT AAC GAA GAA GCC CA-3′/ qP20-p: CGG TTT ACT CGC GAC AAG CTG CTC) (size of PCR product = 109 bp) based on the *p20* gene [19]. RT-qPCR was performed using a Bestar^®^ DBI Taqman PCR Reagent Kit (DBI Bioscience, Shanghai, China) according to the manufacturer’s instructions. To calculate CTV copy number, the conventional PCR product of CTV using the primer set cquctv9/cquctv10 (size of PCR product = 424 bp) [19] was identified by 10 g·L^−1^ agarose gel electrophoresis and purified by DNA Gel extraction kit (TransGen, China). The purified PCR product was inserted into a pEASY-T1 vector (TransGen Biotech, China) to build a recombinant cloning plasmid. After Sanger sequencing, a recombinant plasmid containing cloned CTV cDNA was used as a template to construct standard curves. The copy number of each sample was calculated by the Bio-Rad CFX Manager Software v1.6 (Bio-Rad, Hercules, CA, USA). After qPCR, cDNA samples that yield Ct values ranging from 25.52 to 38.48 were confirmed using Uni-f/r primer through conventional PCR. Visual evaluation of bands obtained by conventional PCR in agarose gel indicated those cDNA samples with Ct ≤ 35, which were considered CTV-positive (i.e., producing a clear band in agarose gel; see Figure S1) and used for subsequent analyses. Samples with Ct values ≥ 37 not generating bands in PCR gels were considered CTV-negative. To avoid errors, samples with intermediate Ct values within 35 to 37 without a clear specific PCR band were not used in further analyses (Figure S1, Table S2). To decrease the concentration difference of cDNA among different samples, CTV titers were assessed using the following formula referenced to the study of Ruiz-Ruiz et al. [46] in CTV detection:

(Copy number of CTV *p20* gene) / Titer of *D. citri* or citrus cDNA per µL.

#### 2.6.3. CTV Genome Detection

Adults of *D. citri* were collected from the CTV-positive “Shatangju” mandarin plants. RNA from *D. citri* were extracted individually. Based on the qPCR-derived calculation of CTV titers in these cDNA samples, a pool of 30 *D. citri* RNA samples yielded RT-qPCR-derived Ct values between 20 and 22 (i.e., relatively high CTV titers) was sequenced using Illumina HiSeq (Illumina, San Diego, CA, USA). The CTV sequences from *D. citri* were reassembled by mapping the obtained reads using Bowtie2 v.2.2.6 [47] and CLC Genomics Workbench v.11 (CLC Bio, Denmark) onto the reference CTV isolate genome DQ151548 (19,252 nt) from Chongqing, China. Gaps in the generated draft genome were filled in using *de novo* assembly with Velvet v.1.2.10 [48], and by recovering any missing sections using cDNA from five *D. citri* individuals as source material for conventional PCR amplification followed by Sanger sequencing of amplicons. Gene prediction within the CTV genome was performed with ORF Finder from NCBI (National Center for Biotechnology Information), RAST server (http://rast.nmpdr.org/, accessed on 12 August 2019) [49], and from references to published CTV genomes. The obtained genome was analyzed with BLAST in NCBI.

#### 2.6.4. Enzyme-Linked Immunosorbent Assays (ELISA)

To further confirm the presence of CTV in citrus and orange jasmine plants, prior to and after *D. citri* transmission, we used ELISA to detect the presence of CTV capsid protein [18,50] via a direct triple antibody sandwich protocol (TAS-ELISA). The capture antibody (Catalog No: CAB 78900; Lot No: 00805) specific to the CTV capsid protein was purchased from Agdia Company (Elkhart, Indiana, USA). ELISA sample extracts were prepared in General Extract Buffer (tissue:buffer = 1:10) from three young leaf midribs (each recorded as a separate repetition) from three different shoots, respectively. The specific capture antibody of CTV was diluted with carbonated coating buffer (1.59 g sodium carbonate and 2.93 g sodium bicarbonate 2.93 g in 800 mL water, adjusted to 1 L with water) at a ratio of 1:100. The CTV detection antibody (A: 00832) and Alk phos enzyme conjugate (B: 00815) were mixed with ECI buffer (add 2 g bovine serum albumin and 20 g polyvinylpyrrolidone MW 24–40,000 to 1000 mL PBST) as suggested by manufacturer’s instructions. PNP substrate solution was used after the detection step. CTV-negative and positive samples can usually be visually discerned based on intrinsic differences in the color intensity in ELISA, detectable by absorbance with a microplate reader (*OD*_405_). Any samples with an OD value higher than 2x the average *OD* of healthy samples were considered “positive” [51].

### 2.7. Statistical Analyses

Data were analyzed and plotted using R (2016) v.3.0.0. Because of relatively low numbers of replications precluding assumptions over data distribution, non-parametric analyses were performed as follows: multifactorial analyses were performed with Kruskal–Wallis comparison tests followed by Dunn’s multiple comparison test (*p-*value < 0.05).

## 3. Results

### 3.1. CTV Orchard Surveys

CTV was detected in 90% (18/20) of “Shatangju” plants, 100% (20/20) of sweet orange samples, and 100% (20/20) of “fingered citron” trees sampled at Qingyuan, Ganzhou, and Zhaoqing, respectively. Conventional PCR of *D. citri* samples with the primer set Uni-f/r detected CTV in 40% (8/20) of samples collected from “Shatangju” trees (Figure S2A), 65% (13/20) of samples from sweet orange trees (Figure S2B), and 95% (19/20) of *D. citri* samples from “fingered citron” trees (Figure S2C). The purified amplicons (822 bp) amplified by RT-PCR were sequenced. Online BLASTn searches of the amplicon sequences on GenBank revealed the highest identity (99%) to the CTV isolate CT11A (JQ911664.1) from Chongqing, China.

Average detection ratios and titers of CTV by RT-qPCR (defined as the ratio of positive/(all samples) ± standard error (SE) followed by relative CTV copy numbers (CN)) in 10 late 4th instars, 10 newly molted 5th instars, 10 late 5th instars, and 10 newly emerged adults of psyllids collected from the CTV-positive trees in the “fingered citron” orchard were 96.67% ± 3.33% (CN = 8739.10 ± 678.02), 96.67% ± 3.33% (CN = 8688.66 ± 680.62), 100% (CN = 9041.85 ± 750.30), and 100% (CN = 9145.66 ± 741.01), respectively. Statistical analysis indicated that the percentage of CTV-positive psyllids and relatively high CTV titers were not influenced by molting nor emergence.

### 3.2. Genetic Characterization of CTV in D. citri

**Figure 2:**
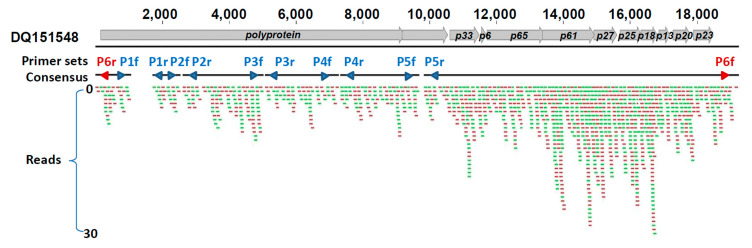
Illumina HiSeq reads coverage by mapping cDNA from *Citrus tristeza virus* (CTV)-positive *Diaphorina citri* onto a reference CTV isolate DQ151548. All reads are 100 bp long, obtained from sequences retrieved from *Diaphorina citri* collected in Guangdong, China. The numbers on the top are nucleotide genome length (nt), and the arrows and letters represent Sanger-sequenced amplicons from 5 PCR primer sets (P1–P5) used to span and bridge the genome, and one primer set (P6) used to suggest a linear structure for CTV genome. “Reads” area span forward reading frames (in red) and reversed reading frames (in green).

Next-generation sequencing (NGS) of CTV-positive *D. citri* returned 1.47 × 10^8^ 100-bp reads. Mapping reads to the reference CTV GenBank isolate DQ151548 (19,252 nt) yielded an average coverage of 16.58× (range from 0.00× to 37.00×) from 1973 mapped reads (0.005% from the total 3.87 × 10^7^ reads). The total consensus length was 17,904 nt, covering 93.24% of the reference genome but leaving five gaps (Figure 2), all within the CTV *polyprotein* gene. Thus, the genome could not be fully reconstructed by *de novo* assembly and was completed using sequences from five PCR amplicons (Figure 2). Single nucleotide polymorphisms (SNPs) (from 0.58% to 3.61%) were found within the gaps of the different individual *D. citri* RNA samples across five genome regions. Based on this sequence data, the genome was estimated to be 19,252 nt long and was named “CTV isolate FN08” (GenBank accession number of MK491895). The CTV-FN08 genome encoded the 11 ORFS observed in other published CTV genomes, indicating the genome was complete. Online GenBank BLASTn searches indicate CTV-FN08 genome is most similar to CTV isolates T318A (DQ151548), CT11A (JQ911664.1), and SG29 (KC748392), with measured identities of 98.50%, 97.97%, and 97.75%, respectively.

### 3.3. CTV Persistence Period Inside D. citri

**Figure 3:**
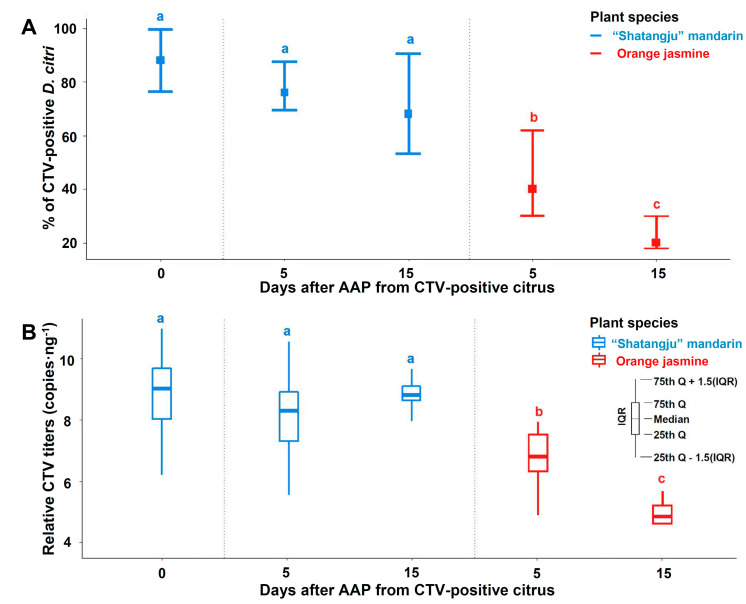
Persistence period of *Citrus tristeza virus* (CTV) in *Diaphorina citri* on different host plant species, as detected by RT-qPCR. (**A**) Incidence of CTV-positive *D. citri*; each treatment (N = 12 or 8 plants) contained 10 *D. citri*. The same letter indicates no significant difference by non-parametric Kruskal–Wallis. (**B**) Box plots of obtained relative titers of CTV in total extracted cDNA (i.e., log-transformed copy numbers of the CTV *p20* gene in *D. citri* cDNA). Non-overlapping error bars between the box plots indicate statistical significance. Legend: “Shatangju” mandarin: C*itrus reticulata* Blanco “Shatangju”; orange jasmine: *Murraya paniculata* (L.) Jack.; AAP: acquisition access period; Q = percentile; IQR = interquartile range; 0 day: testing the psyllids immediately after the end of AAP.

Figure 3 illustrates the acquisition efficiency of CTV by *D*. *citri* and the persistence of the virus inside adults after they were transferred from CTV-positive “Shatangju” mandarin plants to CTV-negative “Shatangju” mandarin plants or orange jasmine plants. The mean percentage of CTV-positive *D*. *citri* adults was 85.00% ± 1.23% (85/100) at the time psyllids were transferred to healthy plants (day 0). The RT-qPCR indicated that 72.50% ± 1.01% and 65.00% ± 2.59% of *D. citri* remained CTV-positive following 5 and 15 days of post-AAP, respectively, on “Shatangju” mandarin plants, but these values taken between two detection times were not significantly different. In contrast, the incidence of CTV-positive adults and their estimated CTV titers decreased significantly after 5 (32.5% ± 7.01% and 1158.78 ± 165.12) and 15 days (5.00% ± 2.67% and 161.53 ± 43.99) following the post-AAP, once they were placed on CTV-negative orange jasmine plants (Figure 3).

### 3.4. Transmission of CTV by D. citri to Mandarin, Lemon, and Orange Jasmine Plants

**Figure 4:**
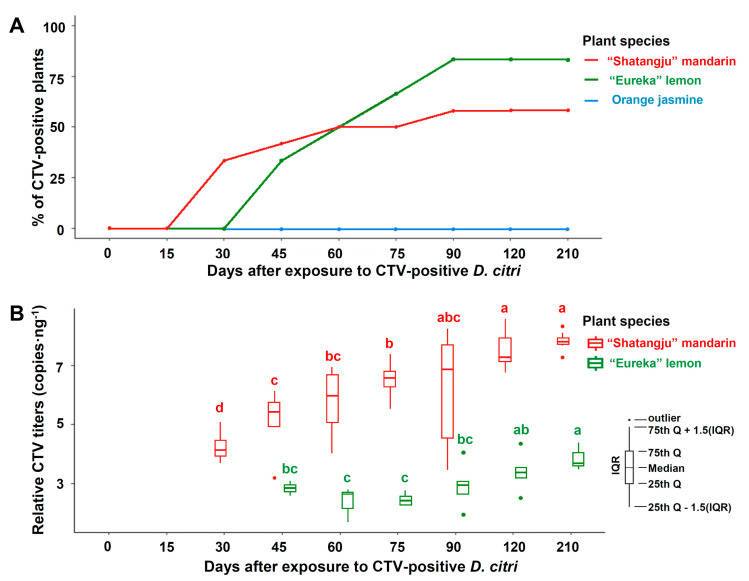
Incidence (**A**) and relative titers (**B**) of *Citrus tristeza virus* (CTV) in three host species after 5-day inoculation access period (IAP) upon exposure to CTV-positive *Diaphorina citri*. Values in (**B**) are log-transformed copy numbers of the CTV *p20* gene from plants/ng of total citrus cDNA. Non-overlapping error bars between the box plots indicate statistical significance. The same letter indicates no significant difference by non-parametric Kruskal–Wallis. Legend: “Shatangju” mandarin: C*itrus reticulata* Blanco “Shatangju”; “Eureka” lemon: *Citrus* × *limon* var. *limon* (L.) Burm. f.; Orange jasmine: *Murraya paniculata* (L.) Jack.; Q = percentile; IQR = interquartile range; 0 days: testing the plants immediately after 5-day IAP by CTV-positive *D. citri*.

Figure 4A shows the percentages of “Shatangju” mandarin, “Eureka” lemon, and orange jasmine plants detected to be CTV-positive by RT-qPCR and ELISA results after 5 days of exposure to batches of 10 CTV-positive adult *D. citri*. Thirty and 90 days after the 5-day IAP exposure to CTV-positive *D. citri*, 33.33% (4/12) and 58.33% (7/12), respectively, of the “Shatangju” mandarin plants were CTV-positive. The virus was not detected in “Eureka” lemon plants 30 days after the IAP. However, 33.33% (2/6) and 83.33% (5/6) of the plants were CTV-positive at 45 and 90 days, respectively, after IAP (Figure 4A). Infection by CTV in “Shatangju” mandarin and “Eureka” lemons was detected by RT-qPCR 210 days after IAP and further confirmed by conventional RT-qPCR and ELISA (Table S3). After 5 days IAP of CTV-positive *D*. *citri*, the eight orange jasmine plants remained CTV-negative for at least 210 days. The virus was not detected in control plants (“Shatangju” mandarin, “Eureka” lemon, and orange jasmine plants) after they were exposed to CTV-negative *D*. *citri* adults for 5 days.

The results from the RT-qPCR indicated that the CTV titers increased significantly over time in the “Shatangju” mandarin plants (*F* = 7.4590; *df* = 6; *p* = 0.0001) and “Eureka” lemon plants (*F* = 4.3090; *df* = 5; *p* = 0.0094) after 5 days of IAP exposure to CTV-positive *D. citri*, and the CTV titers of CTV-positive “Shatangju” mandarin plants were significantly higher than those of the CTV-positive “Eureka” lemons (Figure 4B).

The symptoms observed in crown diameter, plant height, leaf, and stem morphology did not differ significantly between CTV-positive and CTV-negative “Shatangju” mandarin plants. However, CTV-positive “Eureka” lemon plants had significantly smaller crown diameters compared to CTV-negative counterparts (35.17 ± 2.08 cm vs. 50.80 ± 4.18 cm (*p* = 0.0119), respectively) and were significantly shorter (73.90 ± 1.84 cm vs. 90.13 ± 4.33 cm (*p* = 0.0119), respectively) (Figure S3A). Compared to the negative controls of “Eureka” lemon plants, the symptoms of the CTV-positive “Eureka” lemon plants included reduced growth of the young shoots, and yellowing, curling, reduced growth, and luster of the mature leaves. The CTV-positive lemon plants exhibited typical symptoms of rapid decline from 120 to 210 days after the IAP (see symptoms in Figure S3B–D).

## 4. Discussion

Aphids use piercing mouthparts (stylets) to penetrate the plant vascular systems and access sugary fluids in the phloem. This is how viruses are acquired and transmitted during feeding [52]. CTV is acquired and transmitted in a semi-persistent non-circulative manner by several aphid species, including the most important species *Aphis aurantii*, *A. citricidus*, *A. gossypii,* and *A. spiraecola* [26,53]. It was reported that CTV could also be vectored by *Ferrisia virgata* (Cockerell) on lime trees in Africa [54]; however, the reported transmission was based on a single experiment requiring further confirmation. There have been no published reports of the acquisition and transmission of CTV by other phloem-feeding sternorrhynchans, such as psyllids, which feed on *Citrus* and their hybrids. It should be mentioned that *D*. *citri* is often present in citrus plantations where CTV occurs.

Reported CTV acquisition intervals and rates of transmission by aphids are highly variable. For example, *A. citricidus* is regarded as the most effective vector of CTV to sweet orange and Mexican lime trees (*C*. × *aurantiifolia* var. *aurantiifolia* (Christm.) Swingle) [55]. However, the reported AAP for *A. citricidus* to acquire CTV from sweet oranges and pomelo crops ranges from 1 to 24 h, while the exposure duration necessary for transmission ranges from 30 min to 100 h [1,14,21,22]. Moreover, CTV transmission rates by single and multiple (3 to 100) *A. citricidus* ranged from 0.0% to 66.6% and from 0% to 100%, respectively, which is also related to the isolates of CTV [24,55,56,57]. High acquisition rates of CTV by aphids are a necessary precondition for the effective transmission of CTV, as the virus would not multiply within the insect [1,14,21,22].

Recently, a study revealed that CTV was the most abundant virus in Florida *D. citri* populations by high-throughput sequencing [58]. The results in this study indicate a high prevalence (40% to 100%) of CTV among *D. citri* collected from orchards. Moreover, the CTV transmission by groups of 10 CTV-positive *D*. *citri* adults led to 58.33% detection ratios of CTV-positive rate in “Shatangju” mandarin and 83.33% in “Eureka” lemon. Moreover, NGS sequencing suggests the CTV genome retrieved from positive *D. citri* samples was found unbroken.

Although we observed no significant differences between the overall aspect of CTV-positive “Shatangju” mandarin plants and CTV-negative plants, obvious symptoms such as decline (without stem pitting) were observed in positive “Eureka” lemon plants. This could be related to literature claims that resistance in the scions and rootstock relate to different plant virus strains or plant species [41,42,59]. In addition, previous research indicated that the expression, intensity, and severity of the symptoms in citrus depend on the specific infecting CTV strain or a mixture of CTV isolates [2,60]. The CTV strain used in our study was similar to the CTV isolates of the “VT-Asian” subtype [17,61]. The VT genotype viruses are regarded as mild isolates (i.e., symptomless for most citrus) or seedling yellows isolates resulting in symptoms characterized by stunting, small pale or yellow leaves, and a reduced root system in sour orange, grapefruit, or lemon seedlings [2,62,63]. We suppose this is the reason why stem pitting, the most typical symptom of CTV infection in some citrus species, was not observed during the research.

The estimated CTV titers decreased significantly after *D. citri* was transferred from CTV-positive “Shatangju” mandarin trees to orange jasmine plants, while CTV can persist within *D. citri* for some time following the potential transmission to healthy “Shatangju” mandarin plants. In addition, once CTV is acquired by 4th instars, it will persist in the psyllids through nymphal molts and eclosion of 5th instars to adults, suggesting that CTV is transmitted by *D. citri* not in a “semi-persistent” manner as with the aphid vectors of this pathogen. Our observations, albeit preliminary, indicate that the transmission efficiency of the virus by the CTV-positive psyllids to previously healthy “Shatangju” mandarin plants increases with the duration of feeding by the psyllids. Thus, CTV might be persistent but not propagative in *D. citri*, and the mechanisms of CTV transmission require further clarification.

Furthermore, the persistence of detectable CTV within *D. citri* was influenced by the host plant species. The CTV titers of CTV-positive *D. citri* transferred to healthy “Shatangju” host plants did not recede after 15 days (Figure 3), possibly indicating that fresh sap from a suitable host may be necessary for the persistence of CTV in *D. citri* for 15 days or more. Direct evidence of transmission comes from healthy host plants acquiring the virus from positive *D. citri*. The efficiency of the potential CTV transmission was also influenced by host plant species, which suggests further factors (e.g., viral strains and particular aspects of *D. citri* biology) might influence CTV transmission in the field, still warranting further study.

## 5. Conclusions

Our study demonstrates that the Asian citrus psyllid, *D. citri*, can acquire CTV with relatively high efficiency and is capable of transmitting the virus to two citrus species. The results in this preliminary report on CTV acquisition and transmission by *D. citri* were confirmed by different approaches (whole-genome sequencing, conventional PCR, RT-qPCR, and ELISA) relying on detection of viral nucleic acid and capsule proteins, now awaiting further controlled transmission studies and field observations. These findings may raise concerns about the *D. citri* psyllid as a vector related to two important citrus pathogens.

## Data Availability

The data presented in this study are available in the Supplementary Materials. CTV sequence obtained in this study has been deposited in GenBank.

## Supplementary Materials

The following are available online at https://www.mdpi.com/article/10.3390/insects12080735/s1. Figure S1: Sensitiveness of detection of *Citrus tristeza virus* (CTV) cDNA in host samples through conventional PCR with the universal primer set Uni-f/r. Figure S2: *Citrus tristeza virus* (CTV) detection in cDNA extractions of *Diaphorina citri* from orchards in Qingyuan, Guangdong (A), Ganzhou, Jiangxi (B), and Zhaoqing, Guangdong (C), P.R. China. Figure S3: Representative pictures of symptoms of *Citrus* × *Limon* plants after 120 days exposure to CTV-positive *Diaphorina citri* population in comparison to healthy plants after exposure to the CTV-negative psyllids, Table S1: Different species of host plants used in each experiment about CTV acquisition, persistence, and transmission by *Diaphorina citri*. Table S2: Viral load thresholds for the detection of *Citrus tristeza virus* (CTV) in *Diaphorina citri* cDNA samples used in this study, from correlating RT-qPCR quantitative estimations with a visual band detection by PCR. Table S3. ELISA readings of *Citrus tristeza virus* (CTV) capsid proteins in host plants *Citrus reticulata* and *C.* × *Limon* detected at 0 d and 210 days after inoculation access period (IAP) by exposure to CTV-positive *Diaphorina citri.* Supplementary data: Excel file with the data presented in Figure 3 and Figure 4.
