# Supplementary material for: Preliminary Report on the Acquisition, Persistence, and Potential Transmission of Citrus tristeza virus by Diaphorina citri
Source: Insects. 2021 Aug 17;12(8):735. doi: 10.3390/insects12080735 (PMC8396932; doi:10.3390/insects12080735)
Supplement: Supplementary file 1 [file insects-12-00735-s001.zip › Supplementary Tables and Figures.pdf]

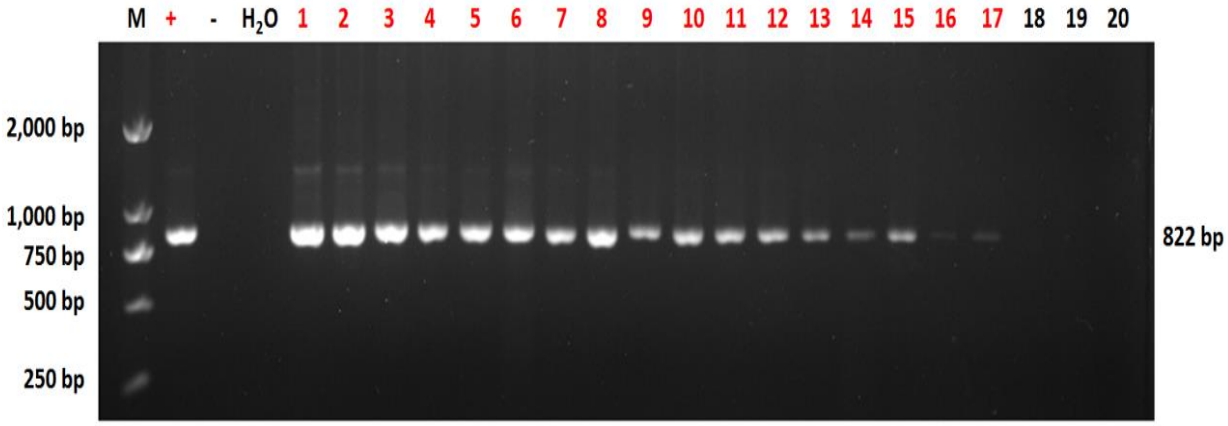

Figure S1. Sensitiveness of detection of *Citrus tristeza virus* (CTV) cDNA in host samples through conventional PCR with the universal primer set Uni-f/r. The primers were designed based on *p27* gene of CTV using Primer3web v.4.1.0. Cycle threshold values of each sample are indicated in Table S2, tested by Taqman qPCR based on CTV *p20* gene (Liu et al. 2008). Numbers in red indicate CTV-positive samples.

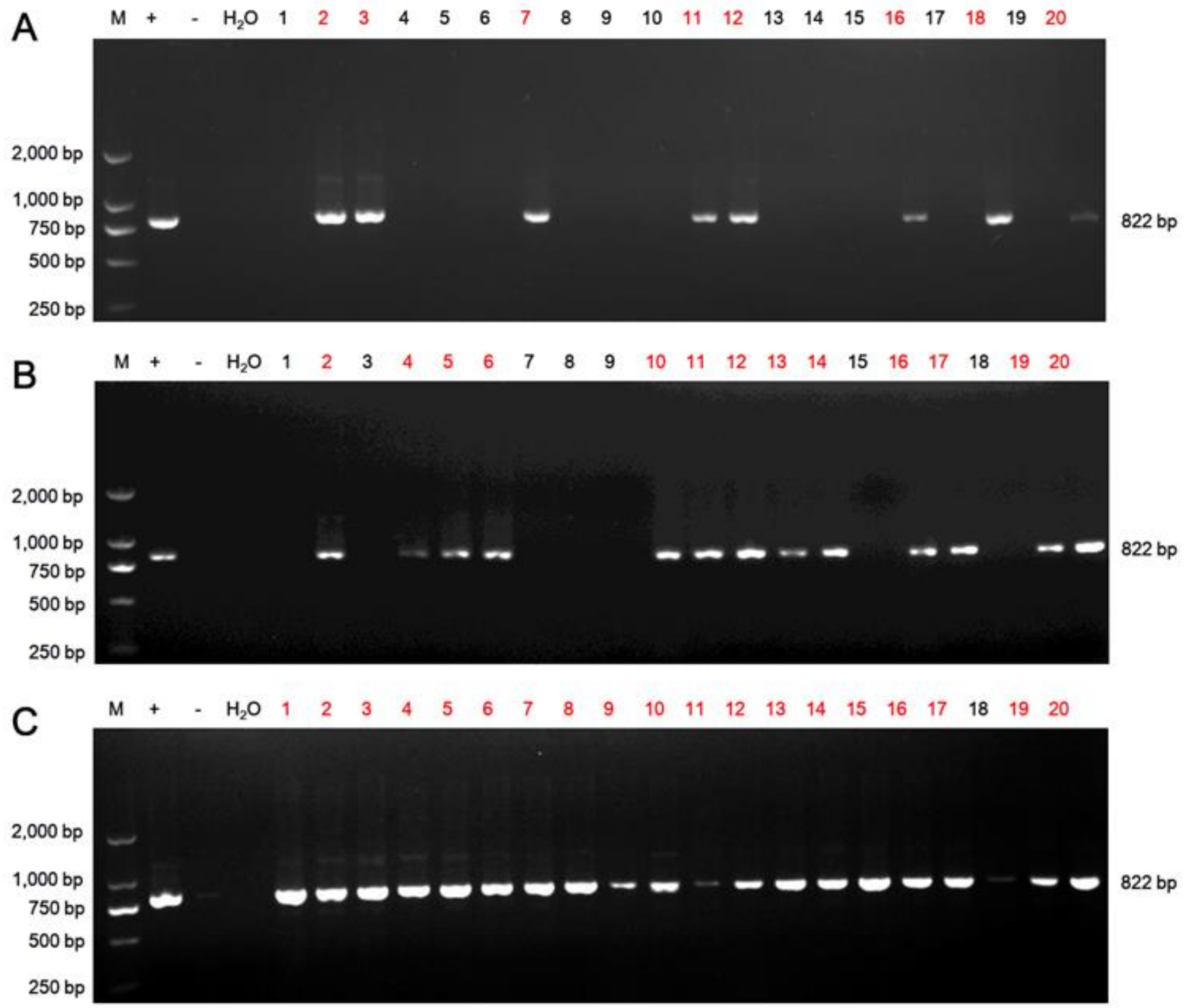

Figure S2. *Citrus tristeza virus* (CTV) detection in cDNA extractions of *Diaphorina citri* from orchards in Qingyuan, Guangdong (A), Ganzhou, Jiangxi (B), and Zhaoqing, Guangdong (C), P.R. China. Primer set Uni-f/r was designed based on *p27* gene of CTV (Liu et al. 2008) using Primer3web v.4.1.0. Numbers in red indicate CTV-positive samples.

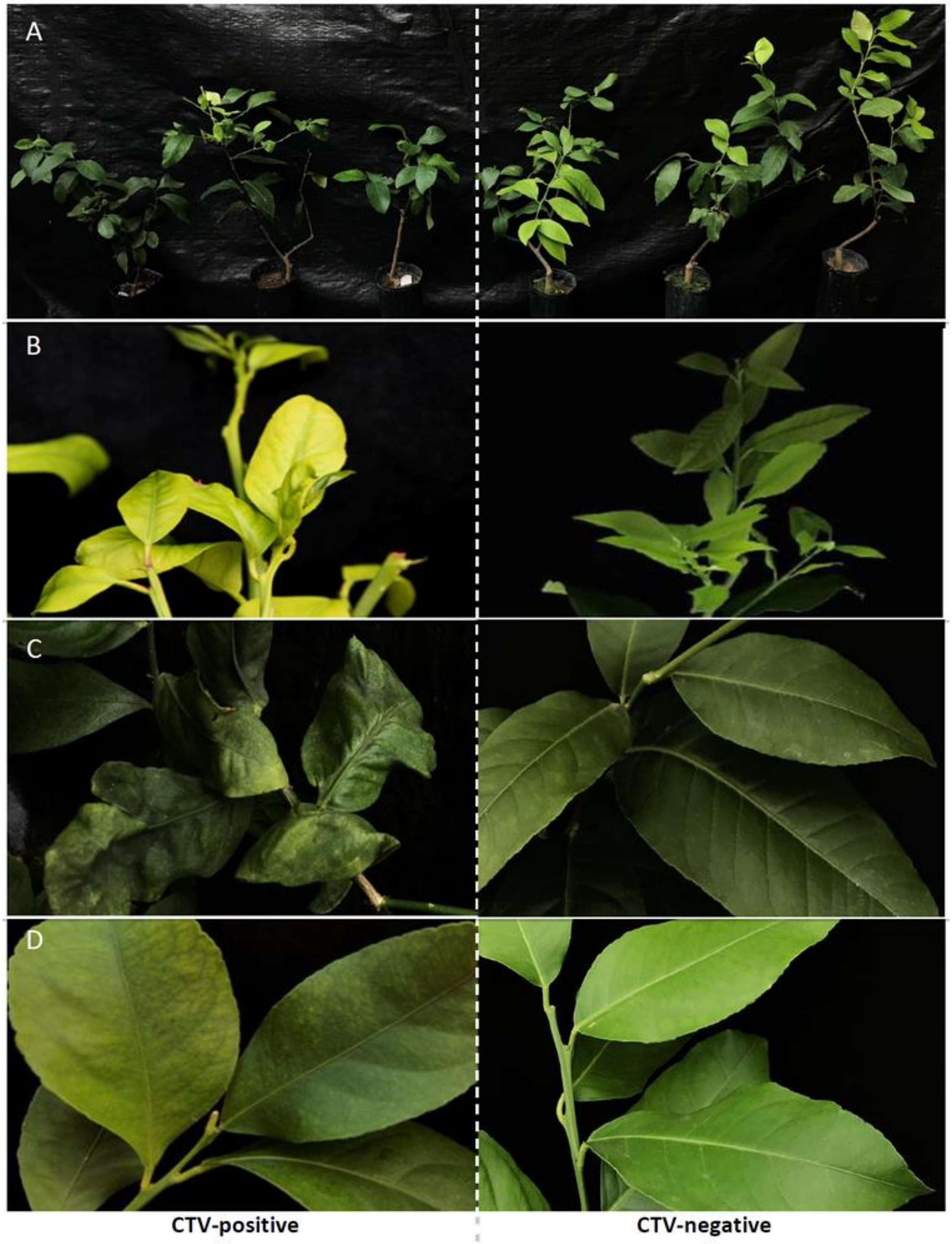

Figure S3. Representative pictures of symptoms of *Citrus tristeza virus* (CTV) infection in *Citrus* × *Limon* plants after 210 days exposure to CTV-positive *Diaphorina citri*, in comparison to healthy plants after exposure to the CTV-negative psyllids. CTV-positive plants are on the left-hand side, while CTV-free plants are shown on the right-hand side. (A) Crown diameter and plant height; (B) Young shoots; (C) Leaf shapes; (D) Mature leaf colors.

Table S1. Host plants with different species used in each experiment about CTV acquisition, persistence and transmission by *Diaphorina citri* [a]

Experiment details	Plant species		No. of host plants	Purposes of the experiment
	English name	Latin name		
Acquisition (Orchard surveys)	'Shatangju' mandarin	*Citrus reticulata* Blanco 'Shatangju'	20	To determine whether *D. citri* can carry CTV universally to the three most common orchard species infected with CTV
	'fingered citron'	*C. medica* L. var. *sarcodactylis* Swingle	20	
	Sweet orange	*Citrus* × *aurantium* var. *sinensis* L.	20	
Acquisition (Laboratory condition)	'Shatangju' mandarin	*Citrus reticulata* Blanco 'Shatangju'	10 (CTV+ *D. citri*), 3 (CTV- *D. citri*)	Further investigate the ability of *D. citri* obtaining CTV, and provide high CTV-positive or -negative psyllids samples for the next experiment
Persistence in *D. citri* (Laboratory condition)	'Shatangju' mandarin	*Citrus reticulata* Blanco 'Shatangju'	12 (5-day IAP), 8 (15-day IAP)	The persistence of CTV in *D. citri* were checked considering the effects between two preferential host species of *D. citri*
	Orange jasmine	*Murraya paniculata* (L.) Jack	8 (5-day IAP), 8 (15-day IAP)	
Transmission of CTV by *D. citri* (Laboratory condition)	'Shatangju' mandarin	*Citrus reticulata* Blanco 'Shatangju'	12 (CTV+ *D. citri*), 3 (CTV- *D. citri*)	Transmission efficiency of CTV by *D. citri* were described under laboratory conditions using three species of Rutaceae family with different tolerance to CTV
	Orange jasmine	*Murraya paniculata* (L.) Jack	8 (CTV+ *D. citri*), 3 (CTV- *D. citri*)	
	'Eureka' lemon	*Citrus* × *limon* var. limon (L.) Burm. f.	6 (CTV+ *D. citri*), 3 (CTV- *D. citri*)	

[a] Legend: CTV+: CTV-positive; CTV-: CTV-negative; IAP: inoculation access periods.

Table S2. Viral load thresholds for the detection of *Citrus tristeza virus* (CTV) in *Diaphorina citri* cDNA samples used in this study, from correlating RT-qPCR quantitative estimations with a visual band detection by PCR [a]

Sample number	Ct value	Relative titer	Conventional PCR band detection
1	25.49 ± 0.04	6738.40 ± 10.25	+
2	25.88 ± 0.50	5801.12 ± 8.36	+
3	26.46 ± 0.04	4026.16 ± 8.55	+
4	27.06 ± 0.05	2794.89 ± 20.16	+
5	27.55 ± 0.02	1339.57 ± 7.89	+
6	27.84 ± 0.11	1137.76 ± 8.32	+
7	28.50 ± 0.04	994.22 ± 9.64	+
8	29.17 ± 0.01	708.57 ± 7.96	+
9	29.34 ± 0.01	578.38 ± 6.10	+
10	29.69 ± 0.08	441.29 ± 3.59	+
11	30.64 ± 0.04	247.78 ± 2.91	+
12	31.12 ± 0.05	174.28 ± 1.06	+
13	31.94 ± 0.05	105.55 ± 0.86	+
14	32.97 ± 0.06	58.92 ± 0.33	+
15	33.81 ± 0.09	29.67 ± 0.24	+
16	34.90 ± 0.05	16.53 ± 0.34	+
<u>17</u>	<u>35.88 ± 0.05</u>	4.84 ± 0.26	+
<u>18</u>	<u>36.86 ± 0.10</u>	1.51 ± 0.08	-
19	37.86 ± 0.06	0.30 ± 0.04	-
20	38.48 ± 0.02	0.18 ± 0.01	-
Positive	27.50 ± 0.04	1113.18 ± 7.41	+
Negative	37.49 ± 0.07	0.46 ± 0.07	-
ddH_2O	N/A	N/A	-

[a] Twenty samples were selected with cycle threshold (Ct) values (*p20* of CTV) ranging from 25.49 to 38.48; means are presented with standard deviations; *D. citri* collected from healthy *Murraya paniculata* were used as negative controls. Samples with values underlined fall in the ambiguous intermediate band detection range, thus were not used in further analyses. qPCR was performed using a DBI Taqman PCR Reagent Kit (Applied Biosystems) using the primer set and probe of qP20-f/r/p. qPCR was performed in three independent assays of each cDNA. Relative titers of CTV=copy numbers of CTV *p20* gene/ ng of total *D. citri* cDNA. Conventional PCR was performed using Uni-f/r primer set designed in this study based on *p27* gene of CTV using Primer3web v.4.1.0, the bands on the gel were indicated in Figure S1. N/A: not available.

Table S3. ELISA readings of *Citrus tristeza virus* (CTV) capsid proteins in host plants *Citrus reticulata* and *C.* ×*Limon* detected at 0 d and 210 days after Inoculation Access Period (IAP) by exposure to CTV-positive *Diaphorina citri* [a]

Plant genotype	Specimen ID	0 d			210 d		
		OD_{405}	S/N	Test results	OD_{405}	S/N	Test results
C. reticulata	R1	0.059	1.11	-	0.319	6.93	+
	R2	0.067	1.26	-	0.221	4.80	+
	R3	0.055	1.04	-	0.283	6.15	+
	R4	0.063	1.19	-	0.185	4.02	+
	R5	0.067	1.26	-	0.269	5.85	+
	R6	0.065	1.23	-	0.220	4.78	+
	R7	0.058	1.09	-	0.284	6.17	+
	R8	0.059	1.11	-	0.063	1.37	-
	R9	0.070	1.32	-	0.063	1.37	-
	R10	0.064	1.21	-	0.064	1.39	-
	R11	0.063	1.19	-	0.076	1.65	-
	R12	0.056	1.06	-	0.064	1.39	-
	CK(+)1	0.121	2.28	+	0.310	6.74	+
	CK(-)1	0.053	1.00	-	0.046	1.00	-
C. ×*Limon*	L1	0.064	1.49	-	0.102	2.32	+
	L2	0.077	1.79	-	0.100	2.27	+
	L3	0.057	1.33	-	0.105	2.39	+
	L4	0.070	1.63	-	0.091	2.07	+
	L5	0.067	1.56	-	0.103	2.34	+
	L6	0.070	1.63	-	0.075	1.70	-
	CK(+)2	0.113	2.63	+	0.265	6.02	+
	CK(-)2	0.043	1.00	-	0.044	1.00	-

[a] ELISA sample extracts were prepared in 1X General Extract Buffer (see the buffer formulations guide from Agdia, Inc. at www.agdia.com / info@agdia.com) from three young leaf midribs (each recorded as a separate repetition) from three different shoots. OD_{405}: optical density at 405 nm; CK(+): CTV infected sample from which the virus was purified for the raising of antibodies; CK(-): healthy sample; S/N: *OD* ratio of specimen/negative. When the ratio reached > 2.00, the sample was considered positive.
